# Supplementary material for: MetaRibo-Seq measures translation in microbiomes
Source: Nat Commun. 2020 Jun 29;11:3268. doi: 10.1038/s41467-020-17081-z (PMC7324362; doi:10.1038/s41467-020-17081-z)
Supplement: Supplementary file 10 — Supplementary Data 7 [file 41467_2020_17081_MOESM10_ESM.zip › File2/Confidence_High_Taxonomy/333882_out.krona.html]

Javascript must be enabled to view this page.

members
magnitude
magnitudeUnassigned
count
unassigned
taxon
rank

333882\_out

13

2
13
superkingdom

1239
13
phylum

186801
13
class

13
186802
order

family
541000
13

13
1263
genus

1
40518
species

SRS050998\_contig\_number\_contig-100\_2311.52529

2293148
5

SRS018623\_contig\_number\_14509SRS018656\_contig\_number\_contig-100\_228.76651SRS053214\_contig\_number\_13268SRS077641\_contig\_number\_23038SRS077730\_contig\_number\_contig-100\_2569.103195
species

7
2293209
species

SRS013476\_contig\_number\_29798SRS024132\_contig\_number\_contig-100\_1468.262111SRS065504\_contig\_number\_contig-100\_7.226226SRS1055083\_contig\_number\_11529SRS144183\_contig\_number\_11855SRS147346\_contig\_number\_66665SRS893383\_contig\_number\_contig-100\_29.204155
